# Supplementary material for: Phenotypic Characterization and RT-qPCR Analysis of Flower Development in F1 Transgenics of Chrysanthemum × grandiflorum
Source: Plants (Basel). 2021 Aug 16;10(8):1681. doi: 10.3390/plants10081681 (PMC8398712; doi:10.3390/plants10081681)
Supplement: Supplementary file 1 [file plants-10-01681-s001.zip › plants-1319372-supplementary.pdf]

Supplementary Data

Table S1. Analysis of growth parameters of F_1 transgenic and non-transgenic Chrysanthemum plants exposed to 100%, 80%, 60%, 40%, and 20% irradiance.

Cross Combinations	Treatment	Plant Height (cm)	Diameter of the main stem (cm)	Leaf length (cm)	Leaf Width (cm)	Length:Width Ratio
	A	40.30 ± 3.31 ab	0.65 ± 0.11 a	6.11 ± 0.63 b	5.09 ± 0.54 b	1.20 ± 0.09 b
	B	38.51 ± 3.51 b	0.55 ± 0.09 b	6.61 ± 0.96 ab	5.26 ± 0.64 ab	1.26 ± 0.13 a
	C	38. 82 ± 2.51 b	0.48 ± 0.09 b	6.65 ± 1.05 b	5.37 ± 0.88 b	1.24 ± 0.14 ab
G x R	D	42.81 ± 3.87 a	0.43 ± 0.08 b	6.95 ± 1.18 a	5.61 ± 0.85 a	1.24 ±0.12 ab
	E	40.26 ± 3.27 ab	0.35 ± 0.05 c	6.54 ± 1.01 ab	5.38 ± 0.83 ab	1.22 ± 0.10 ab
	A	48.10 ± 3.31 ab	0.59 ± 0.10 a	6.23 ± 0.68 b	5.03 ± 0.51 b	1.24 ± 0.08 b
	B	36.55 ± 3.51 b	0.47 ± 0.07 b	6.66 ± 1.05 ab	5.31 ± 0.69 ab	1.25 ± 0.11 a
37 x R	C	36. 63 ± 2.51 b	0.50 ± 0.07 b	6.34 ± 1.09 b	5.22 ± 0.83 b	1.21 ± 0.14 ab
	D	50.93 ± 3.96 a	0.48 ± 0.05 b	7.12 ± 1.25 a	5.67 ± 0.91 a	1.26 ±0.16 ab
	E	48.21 ± 3.27 ab	0.37 ± 0.03 c	6.61 ± 1.02 ab	5.45 ± 0.86 ab	1.21 ± 0.12 ab
	A	48.15 ± 3.27 ab	0.62 ± 0.15 a	6.23 ± 0.68 b	5.06 ± 0.53 b	1.23 ± 0.06 b
	B	36.63 ± 3.56 b	0.48 ± 0.06 b	6.66 ± 1.05 ab	5.35 ± 0.72 ab	1.24 ± 0.13 a
37 x G	C	37. 72 ± 2.51 b	0.52 ± 0.08 b	6.73 ± 1.09 b	5.44 ± 0.84 b	1.21 ± 0.15 ab
	D	51.95 ± 3.98 a	0.49 ± 0.07 b	7.12 ± 1.25 a	5.70 ± 0.94 a	1.25 ±0.15 ab
	B	49.28 ± 3.30 ab	0.38 ± 0.03 c	6.61 ± 1.02 ab	5.47 ± 0.89 ab	1.21 ± 0.13 ab

Table S2. Analysis of leaf gaseous exchange parameters of F_1 transgenic and non-transgenic Chrysanthemum plants exposed to 100%, 80%, 60%, 40%, and 20% irradiance.

Cross Combinations	Treatment	LCP/$\mu mol \cdot m^{-2} s^{-1}$	AQY	P_n /$\mu mol \cdot m^{-2} s^{-1}$	G_s/ $\mu mol \cdot m^{-2} s^{-1}$	C_i/ $\mu mol \cdot m^{-2} s^{-1}$	T_r/ $\mu mol \cdot m^{-2} s^{-1}$	L_s/ $\mu mol \cdot m^{-2} s^{-1}$
	A	49.01 ± 0.04 a	0.053± 0.003 a	22.84± 1.41 a	0.65± 0.14 a	831.43 ± 4.54 a	5.42± 0.65 a	0.53 ± 0.02 a
	B	47.89 ± 0.10 b	0.053± 0.001 a	25.26± 2.78 b	0.48± 0.5 ab	846.61 ± 9.29 a	5.29± 0.64 a	0.55 ± 0.05 a
G x R	C	46.59 ± 0.13 c	0.053± 0.002 a	21.71± 0.62 c	0.39± 0.4 bc	815.43 ± 8.31 a	5.05± 0.35 ab	0.57 ± 0.05 a
	D	45.60 ± 0.11 d	0.047± 0.003 b	20.45± 0.51 d	0.24± 0.3 cd	803.38 ± 10.57 a	1.84± 0.34 ab	0.62 ± 0.09 a
	E	31.43 ± 0.34 c	0.042± 0.002 c	18.17± 0.46 e	0.21± 0.3 d	788.38 ± 15.81a	1.55± 0.30 b	0.61 ± 0.08 a
	A	58.08± 0.02 a	0.074 ± 0.005 a	30.86± 1.39 a	0.75 ± 0.09 a	840.42± 5.51 a	5.51 ± 0.65 a	0.62± 0.04 a
	B	56.75± 0.19 b	0.074± 0.003 a	34.31± 2.81 b	0.48 ± 0.3 ab	856.63± 9.43 a	5.33 ± 0.64 a	0.64± 0.05 a
37 x R	C	55.51± 0.16 c	0.074± 0.002 a	29.72± 0.63 c	0.39± 0.5 bc	823.37 ± 8.4 a	5.12 ± 0.41 ab	0.66± 0.05 a
	D	54.66± 0.11 d	0.053± 0.003 b	28.51± 0.52 d	0.30 ± 0.2 cd	813.39 ± 10.68 a	1.88 ± 0.38 ab	0.70± 0.07 a
	E	39.78± 0.33 c	0.051± 0.001 c	26.19± 0.44 e	0.27 ± 0.2 d	800.35 ± 15.94a	1.59 ± 0.30 b	0.69± 0.07 a
	A	60.01 ± 0.02 a	0.076 ± 0.006 a	32.92 ± 1.41 a	0.77 ± 0.12 a	843.48 ± 5.54 a	5.53 ± 0.69 a	0.64 ± 0.04 a
	B	58.85 ± 0.15 b	0.076 ± 0.003 a	36.28 ± 2.84 b	0.52 ± 0.4 ab	858.67 ± 9.47 a	5.36 ± 0.68 a	0.66 ± 0.06 a
37 x G	C	57.58 ± 0.18 c	0.076 ± 0.002 a	31.75 ± 0.66 c	0.41 ± 0.6 bc	825.41± 8.37 a	5.15 ± 0.46 ab	0.68 ± 0.06 a
	D	56.65 ± 0.13 d	0.056 ± 0.003 b	30.55 ± 0.56 d	0.33 ± 0.3 cd	815.42 ± 10.71 a	1.95 ± 0.40 ab	0.72 ± 0.09 a
	E	41.48 ± 0.37 c	0.052 ± 0.002 c	28.21 ± 0.49 e	0.30 ± 0.3 d	802.38 ± 15.97a	1.62 ± 0.33 b	0.71 ± 0.09 a

Table S3. Analysis of chlorophyll fluorescence parameters of F_1 transgenic and non-transgenic Chrysanthemum plants exposed to 100%, 80%, 60%, 40%, and 20% irradiance.

Cross Combinations	Treatment	F_v/F_m	ϕPSII	$F_{v'}/F_{m'}$	ETR	qP	NPQ
G x R	A	0.80 ± 0.006 b	0.597 ± 0.024 a	0.726 ± 0.023 a	87.56 ± 0.101 c	0.783 ± 0.016 a	2.437 ± 0.136 a
	B	0.834 ± 0.008 a	0.623 ± 0.048 a	0.742 ± 0.027 a	88.72 ± 0.117 b	0.795 ± 0.045 a	2.075 ± 0.195 b
	C	0.841 ± 0.011 a	0.636 ± 0.023 a	0.754 ± 0.028 a	90.36 ± 0.193 a	0.811 ± 0.005 a	2.097 ± 0.092 b
	D	0.845 ± 0.006 a	0.615 ± 0.036 a	0.736 ± 0.034 a	87.44 ± 0.087 cd	0.784 ± 0.010 a	2.103 ± 0.166 b
	E	0.849 ± 0.004 a	0.549 ± 0.006 b	0.721 ± 0.021 a	87.31 ± 0.051 d	0.712 ± 0.025 b	2.229 ± 0.254 ab
37 x R	A	0.824 ± 0.008 b	0.620 ± 0.034 a	0.744 ± 0.026 a	94.75 ± 0.110 c	0.812 ± 0.015 a	2.482 ± 0.148 a
	B	0.841 ± 0.009 a	0.637 ± 0.050 a	0.760 ± 0.031 a	95.77 ± 0.116 b	0.824 ± 0.046 a	2.130 ± 0.193 b
	C	0.847 ± 0.012 a	0.639 ± 0.025 a	0.773 ± 0.032 a	97.42 ± 0.194 a	0.839 ± 0.006 a	2.113 ± 0.092 b
	D	0.853 ± 0.008 a	0.614 ± 0.036 a	0.746 ± 0.030 a	94.58 ± 0.085 cd	0.812 ± 0.010 a	1.996 ± 0.160 b
	E	0.859 ± 0.005 a	0.582 ± 0.006 b	0.741 ± 0.016 a	94.42 ± 0.048 d	0.735 ± 0.022 b	2.241 ± 0.258 ab
37 x G	A	0.838 ± 0.010 b	0.628 ± 0.037 a	0.753 ± 0.032 a	95.82 ± 0.113 c	0.817 ± 0.020 a	2.487 ± 0.152 a
	B	0.869 ± 0.012 a	0.642 ± 0.055 a	0.764 ± 0.035 a	96.81 ± 0.119 b	0.830 ± 0.051 a	2.135 ± 0.198 b
	C	0.853 ± 0.015 a	0.631 ± 0.030 a	0.778 ± 0.036 a	98.48 ± 0.198 a	0.843 ± 0.008 a	2.119 ± 0.096 b
	D	0.857 ± 0.011 a	0.620 ± 0.039 a	0.752 ± 0.038 a	95.64 ± 0.093 cd	0.818 ± 0.016 a	2.103 ± 0.166 b
	E	0.853 ± 0.006 a	0.558 ± 0.009 b	0.746 ± 0.021 a	95.52 ± 0.058 d	0.742 ± 0.025 b	2.252 ± 0.263 a
